# Airborne Halophilic and Non‐Halophilic Microbial Communities in an Underground Salt Mine Affected by Seasonal Environmental Fluctuations

**DOI:** 10.1111/1758-2229.70293

**Published:** 2026-02-18

**Authors:** Aleksandra Puławska, Michalina Rachubik, Dominika Drzewiecka, Luciana Albuquerque, Conceiçao Egas, Jolanta Kalinowska, Krzysztof Krawczyk, Maciej Manecki, Camille Locht, Magdalena Kowalewicz‐Kulbat

**Affiliations:** ^1^ Department of Mineralogy, Petrography and Geochemistry Faculty of Geology, Geophysics and Environmental Protection, AGH University of Krakow Krakow Poland; ^2^ Department of Immunology and Infectious Biology Institute of Microbiology, Biotechnology and Immunology, Faculty of Biology and Environmental Protection, University of Lodz Lodz Poland; ^3^ Department of Biology of Bacteria Institute of Microbiology, Biotechnology and Immunology, Faculty of Biology and Environmental Protection, University of Lodz Lodz Poland; ^4^ CNC‐UC – Center for Neuroscience and Cell Biology, University of Coimbra Cantanhede Portugal; ^5^ CIBB – Center for Innovative Biomedicine and Biotechnology, University of Coimbra Cantanhede Portugal; ^6^ Genoinseq – Next Generation Sequencing Unit, Biocant, BiocantPark Cantanhede Portugal; ^7^ Univ. Lille, CNRS, Inserm, CHU Lille, Institut Pasteur de Lille, U1019 ‐ UMR9017 ‐ CIIL ‐ Center for Infection and Immunity of Lille Lille France

**Keywords:** aerosols, halophiles, humidity, microclimate, salt mine, seasonality

## Abstract

The UNESCO‐listed Bochnia Salt Mine in Poland is a heritage site whose galleries remain coupled to surface air, generating warm, humid, salt‐laden summers and cool, dry, ion‐poor winters. Airborne halophilic archaea were recently reported here but their seasonal behaviour remained unknown. We quantified physicochemical parameters and airborne microbes at three underground stations and an outdoor control site. Winter measurements of air samples were compared with previously published summer data from the same transect. Winter relative humidity (~42%–63%) was lower than summer values (~65%–77%). Furthest from the intake shaft, the combined Na^+^ + Cl^−^ aerosol load fell from 2665 to 1280 μg/m^3^ (~52%), and cultivable halophilic archaea dropped from 7164 to 528 colony‐forming units/m^3^ (~93%). Averaged across the underground stations, halophilic archaeal densities declined by at least 13‐fold between seasons. Halophilic richness contracted from seven to two taxa. Only *Halalkalicoccus subterraneus* and *Halococcus hamelinensis* persisted through winter. Non‐halophilic bacterial loads varied by 3.5‐fold but species richness was relatively high. The visitor‐intensive chamber was dominated by skin‐associated bacteria, underscoring anthropogenic control of non‐halophilic communities. This first demonstration of seasonally responsive airborne halophilic archaea shows that their abundance and diversity are strongly influenced by microclimatic water availability in subterranean salt environments.

## Graphical abstract

This study tests seasonal controls on airborne microbes in the UNESCO‐listed Bochnia Salt Mine. In summer, relative humidity exceeded the halite deliquescence threshold, coinciding with higher NaCl aerosol and greater densities of halophilic microbes; in winter, humidity stayed below deliquescence and halophilic microbes declined at least 13‐fold while halophilic archaea richness fell from seven to two taxa, with only *Halalkalicoccus subterraneus* and *Halococcus hamelinensis* persisting. Thus, halophilic archaea track in mine air moisture and saline aerosol loading.

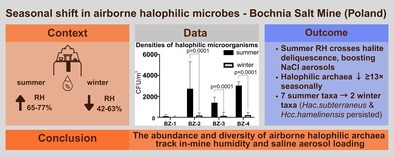

## 1 Introduction

Halophilic microorganisms, including bacteria and archaea, that require elevated concentrations of NaCl have evolved specialised strategies that enable them to withstand concurrent stresses, such as low water activity, desiccation, oxidative damage and chronic nutrient limitation (Ma et al. 2010; Oren 2024). Although their ecology is relatively well understood in surface hypersaline systems, including salt lakes, saline soils and solar salterns (Cycil et al. 2020; DasSarma et al. 2021; Thompson, Kelly, et al. 2021; Thompson, Megaw, et al. 2021), airborne microorganisms living in hypersaline environments have comparatively remained understudied.

Salt mines offer unique opportunities to study extremophiles under natural conditions (DasSarma and DasSarma 2017; Carpa et al. 2014; Megaw et al. 2019; Cockell et al. 2020). Halite can preserve cells and biomolecules for geological timescales, but sharp salinity–humidity gradients impose intense selection pressures (Vreeland et al. 2000; Lowenstein 2012; Jaakkola et al. 2016; Gramain et al. 2011; Schreder‐Gomes et al. 2022). These environments also serve as analogues for extraterrestrial habitability and potential biotechnological applications (Moopantakath et al. 2023; Bader et al. 2018; Thompson and Gilmore 2023; Kowalewicz‐Kulbat et al. 2023; Wu et al. 2022; Bayles et al. 2020). More recently, halophilic microorganisms have also been explored for their potential role in respiratory therapies such as halotherapy (Krawczyk et al. 2022).

Air represents an increasingly recognised vector that connects various habitats. However, the aerobiome of underground salt mines has received scant attention. Early surveys of Polish mines, such as Bochnia, Wieliczka and Polkowice–Sieroszowice, documented predominantly non‐halophilic bacteria and fungi introduced via ventilation air and human traffic (Frączek et al. 2013; Gębarowska et al. 2018; Myszkowska et al. 2019; Górny et al. 2020). Recently, we reported the presence of viable halophilic bacteria and archaea in the atmosphere of the Bochnia Salt Mine, thereby expanding the known dispersal range of halophiles and raising new questions regarding the factors that govern their airborne persistence (Puławska et al. 2025).

In subterranean environments, physicochemical conditions fluctuate with both depth and season, driven by ventilation regimes and external climate. Temperature and, above all, relative humidity (RH) have repeatedly been identified as key determinants of microbial abundance and viability in mine air (Frączek et al. 2013; Górny et al. 2020). No published data is available on the seasonal pattern of halophilic microorganisms; for non‐halophilic bacterial and fungal bioaerosols in the Bochnia Salt Mine, previous year‐round monitoring has already documented clear seasonal patterns in their concentrations and composition (Frączek et al. 2013). For halophiles, organisms exquisitely sensitive to water activity, humidity‐mediated changes in aerosol microhabitats are likely to act as a strong ecological filter. However, to date, no study has resolved the seasonal dynamics of airborne halophilic communities or quantified the relative influence of environmental versus anthropogenic drivers.

In this study, we compared the winter and summer physicochemical parameters and microbial composition, including halophilic archaea, of air sampled from four distinct locations within the Bochnia Salt Mine. We examined the density of airborne halophilic and non‐halophilic microorganisms as a function of distance from the mine entrance, as well as seasonal variations in humidity, temperature, ion concentration and human activity. We prioritised cultivable halophilic microorganisms to secure a resource for bioprospecting novel extremozymes and antimicrobial compounds (Thompson and Gilmore 2023; Kowalewicz‐Kulbat et al. 2023) for future studies and to directly address the public health relevance of salt mines, which operate as a subterranean facility where patients inhale saline aerosols for respiratory benefits (Wasik and Tuuminen 2021; Puławska, Manecki, Flasza, and Styszko 2021).

## 2 Materials and Methods

### 2.1 Sampling

Sampling was conducted in February 2023, following the same strategy and using the same sampling locations as in our previous study carried out in the Bochnia Salt Mine during the summer season (Puławska et al. 2025). Aerosol samples were collected at four locations (BZ‐1 to BZ‐4) along the main airflow path, positioned progressively from the ventilation air entrance (BZ‐1) toward the distal corridor section (BZ‐4). Distances from the air inlet were 0, 1185, 1728 and 2671 m, respectively, with corresponding air velocities of 1.85, 1.72, 0.02 and 0.15 m s^−1^. According to information provided by the mining staff, these airflow velocities remain constant across seasons due to the mechanically regulated ventilation system. This spatial gradient corresponds to the layout previously illustrated in Puławska et al. (2025). During the sampling days, visitor numbers were 497 in the summer and 104 in the winter, representing high and low tourist activity periods, respectively. At each sampling point (BZ‐1, BZ‐2, BZ‐3 and BZ‐4), air temperature and humidity were measured, and the chemical composition of aerosols and microbiological content were analysed. Samples were collected in triplicate. A detailed description of the sampling sites and procedures is provided in Puławska et al. (2025). Briefly, total suspended particulate (TSP) matter from approximately 5 m^3^ of air was collected on quartz filters (Whatman QMA, ϕ25 mm) using battery‐powered personal air samplers (Gilian pumps, Sensidyne, USA). Dreschler samplers were used for wet sampling to determine ion chemical composition. For the sampling of microorganisms, 100 L of air/sample site was aspirated using the impaction method with the MAS‐100 Eco air sampler (Merck) directly onto plates containing Tryptic Soy Agar (TSA; BioMaxima, Lublin, Poland) or *Halobacterium* medium (HBM) containing 5 g/L yeast extract, 5 g/L casamino acids, 1 g/L Na‐glutamate, 2 g/L KCl, 3 g/L Na_3_‐citrate, 20 g/L MgSO_4_·7 H_2_O, 36 mg/L FeCl_2_·4 H_2_O, 360 ng/L MnCl_2_·4 H_2_O, 20 g/L agar and 15%, 20% or 25% NaCl.

The results were compared with those obtained in the summer described in the previous study (Puławska et al. 2025). The summer and winter sampling campaigns were selected to represent the most contrasting environmental conditions in the Bochnia Salt Mine, corresponding to the highest and lowest recorded values of relative humidity and temperature. This approach was designed to capture the maximal seasonal variability in airborne microbial communities rather than to assess interannual fluctuations, as the study focused on the seasonal variability of airborne microbial communities in relation to humidity, temperature and aerosol chemical composition. Other environmental parameters, including oxygen and CO_2_ concentrations and trace gases, were not measured, as the mechanically controlled ventilation system maintains stable air circulation and gas composition throughout the year (Puławska, Manecki, Flasza, and Styszko 2021).

### 2.2 Chemical Analysis of Aerosols

The concentrations of cations (Na^+^, NH_4_^+^, Mg^2+^, K^+^, Ca^2+^, Fe^3+^, Mn^2+^, Al^3+^, Ti^4+^, As^3+^, Ba^2+^, Cr^3+^, Cu^2+^, Mo^6+^, Ni^2+^, Pb^2+^, Zn^2+^) in air samples collected via the wet method were measured using inductively coupled plasma mass spectrometry (ICP‐MS; ELAN 6100; PerkinElmer). The concentrations of anions (F^−^, Cl^−^, Br^−^, PO_4_^3−^, SO_4_^2−^) were determined using ion chromatography (IC; ICS‐1100 Thermo Scientific) as described in Puławska et al. (2025). Total suspended particles (TSP) were measured gravimetrically by weighing the filters without performing size‐fractionated analyses.

### 2.3 Microbial Isolation and Culture Conditions

TSA (15.0 g/L pancreatic digest of casein, 5.0 g/L soy pepton, 5.0 g/L NaCl, 15 g/L agar) and HBM agar plates with 15%, 20% or 25% NaCl were incubated at 21°C, 28°C or 37°C under humid conditions up to 10 days for non‐halophiles and up to 3 months for halophiles. After incubation, the numbers of colony‐forming units (CFU) were calculated and expressed as CFU/m^3^ of air. Based on the morphological characteristics of the colonies growing on TSA or HBM agar, single colonies were cultured on fresh TSA or HBM agar with the same NaCl concentration as the initial plates until the single colonies were obtained. The pure isolates were frozen in 50% glycerol and stored at −80°C until used for molecular, microbiological and biochemical studies.

### 2.4 Identification of Halophilic Microbial Communities by 16S rRNA Gene Sequencing

To identify the isolated strains grown on HBM agar at the species level, genomic DNA was extracted using the Nielsen method (Nielsen et al. 1995), as described (Puławska et al. 2025). The 16S rRNA gene was amplified by PCR. For bacterial isolates the primers used were 27F (5′‐GAGTTTGATCCTGGCTCAG‐3′) and 1525R (5′‐AGAAAGGAGGTGATCCAGCC‐3′). For archaea, the primers used were 21F (5′‐TTCCGGTTGATCCTGCCGGA‐3′) and 1492R (5′‐TACGGYTACCTTGTTACG‐3′). The 16S rRNA PCR products were purified with NZYGelpure (NZYtech) according to the manufacturer's protocol. The samples were stored at −20°C until analysed. The 16S rRNA gene sequence (~700 bp) was determined by Sanger sequencing (Stab Vida, Portugal). Based on the partial 16S rRNA gene sequences, the taxonomic affiliation of each isolate was determined using the online EzBioCloud database (Yoon et al. 2017).

### 2.5 Identification and Biochemical Analysis of Non‐Halophilic Bacterial Communities

Pure isolates of bacteria which grew on TSA plates were Gram stained and identified using the system VITEK 2 COMPACT by Biomerieux based on colorimetric reactions. The VITEK 2 COMPACT system is a fully automated microbial diagnostic tool. Pure bacterial colonies were isolated and suspended in sterile saline with turbidity adjusted to correspond to the 0.5 McFarland standard. For resistance evaluation, specific cards for Gram‐positive (GP), Gram‐negative (GN) and spore‐inducing bacilli (BCP) were employed. This was outsourced to the diagnostic laboratory ‘Mikrografia’ in Krakow. In addition, the VITEK 2 system allowed us to characterise enzymatic activity, sugar metabolism and antibiotic resistance of the isolated bacteria.

### 2.6 Statistical Analyses

Statistical analyses were performed with the GraphPad Prism 7 and STATISTICA 12.0 PL programs. Data are expressed as means ± SD or medians ± interquartile range. Differences between samples were analysed by the variance Kruskal–Wallis non‐parametric test and the Mann–Whitney *U* test. *p* values ≤ 0.05 were considered significant.

## 3 Results

### 3.1 The Physicochemical Characteristics of the Air in the Salt Mine During the Winter Season

**FIGURE 1:**
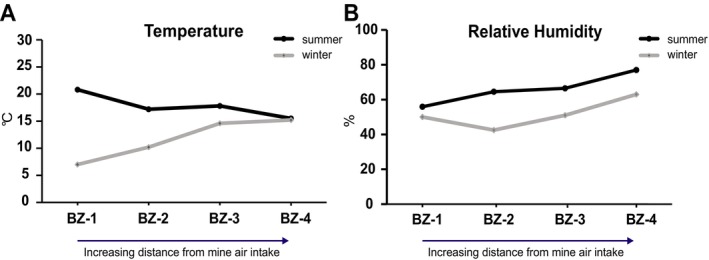
Seasonal variation in air temperature (A) and relative humidity (B) at the four sampling sites BZ‐1 to BZ‐4. Line plots compare values recorded during summer (black lines) and winter (grey lines) at the four different sites.

During the winter, from measurement point BZ‐1 (at the mine entrance) to BZ‐4 (the furthest site), the air temperature increased gradually from 7.0°C to 15.2°C. At the same time, the RH rose from 50.0% to 63.0% (Figure 1). This profile was different from that of the summer season (Figure 1) where, from the entry to the furthest site, the temperature gradually decreased from 20.8°C to 15.5°C, accompanied by an increase in RH from 55.9% to 77.0% (Puławska et al. 2025). As expected, these patterns indicate that in the summer, higher temperatures and lower humidity are observed near the entrance, whereas deeper within the mine, the air becomes cooler and more humid. In the winter, although the temperature and humidity also change with depth, the overall conditions remain cooler and less humid compared to the summer.

**FIGURE 2:**
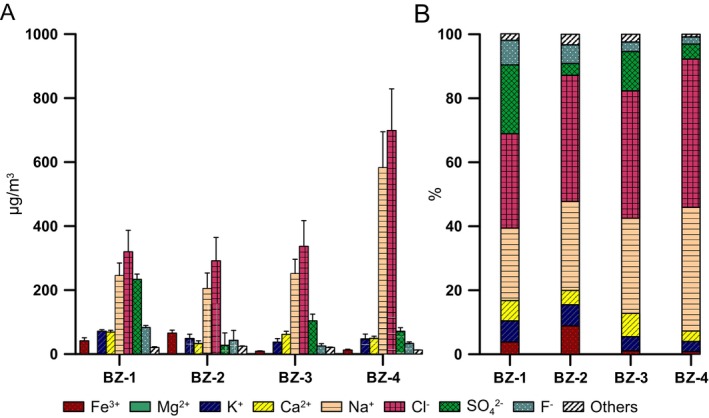
Ion concentrations in the liquid aerosol samples collected at four sampling points (BZ‐1 to BZ‐4). Panel A shows the absolute concentrations in air (μg/m^3^), while Panel B presents the percentage composition of selected ions expressed as relative proportions (%). Elements below the detection limit for all sampling events are not reported.

**FIGURE 3:**
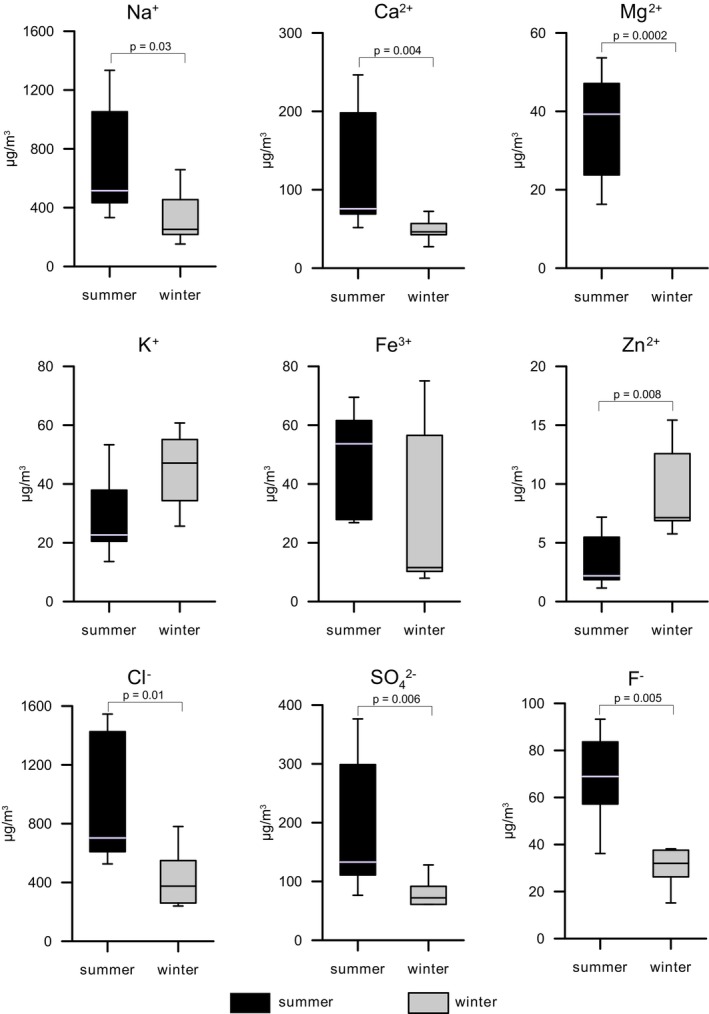
Seasonal variation in the concentration of selected ions in the air summarised at three internal sampling sites within the underground salt mine (BZ‐2, BZ‐3, BZ‐4). Box plots show the distribution of values for summer and winter. Boxes represent the interquartile range, the horizontal line inside the box indicates the median, and whiskers denote the minimum and maximum values. Statistically significant differences between seasons are marked by *p*‐values.

Concerning the ion concentrations, in BZ‐4, the site furthest away from the mine entrance (2671 m), the air contained more NaCl than in the other sampling sites (Figure 2A), like what has been observed in the summer season (Puławska et al. 2025). In the winter samples, sodium and chloride dominated the ion profile, with concentrations increasing from BZ‐1 to BZ‐4 (Na^+^: 246 to 583 μg/m^3^; Cl^−^: 320 to 699 μg/m^3^). Potassium and calcium exhibited inconsistent distributions without a clear spatial trend. Sulphate levels were highest in BZ‐1, dropped sharply in BZ‐2, and then rose slightly at greater distances from the entrance. Fluoride showed a steady decrease with distance, while magnesium and phosphate remained at negligible levels throughout. Iron concentrations peaked in BZ‐2 and declined at the more distant sites (Figure 2B). The concentration of these ions exhibited significant seasonal variation. When combined together, the concentrations of Na^+^, Ca^2+^, Mg^2+^, Cl^−^, SO_4_^2−^ and F^−^ were significantly lower in the winter compared to the summer, while the opposite was observed for Zn^2+^ (Figure 3). For K^+^ and Fe^3+^, no significant differences were observed between the two seasons. Liquid‐phase aerosols (saline droplets), which constituted the predominant aerosol fraction in the studied salt mine, exhibited ion concentrations several hundred times higher than the TSP levels in air, regardless of season.

### 3.2 Microbial Composition of Air Samples

**FIGURE 4:**
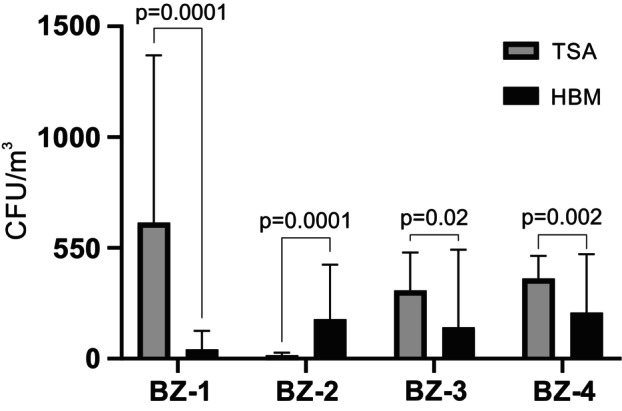
Numbers of colonies of non‐halophilic microorganisms grown on TSA (white bars) and halophilic microorganisms grown on HBM agar containing 15%, 20% or 25% NaCl (black bars). Colonies were counted after growth for 10 days and 3 months, respectively, at 21°C, 28°C or 37°C. The values shown represent the average CFU counts ± standard deviations at all relevant conditions, with three samples counted for each condition.

To study the relative proportions of cultivable halophilic microorganisms compared to other microorganisms in the four sampling sites, all cultivable microorganisms from 100 L of air were grown on Tryptic Soy Agar (TSA) or *Halobacteria* Medium (HBM) agar containing 15%, 20% or 25% NaCl at 21°C, 28°C or 37°C. Combining all temperatures and salt concentrations, BZ‐1, BZ‐3 and BZ‐4 were dominated by CFU counts on TSA, while BZ‐2 was dominated by CFU counts on HBM agar (Figure 4).

### 3.3 Halophilic Microorganism Composition and Identification

**FIGURE 5:**
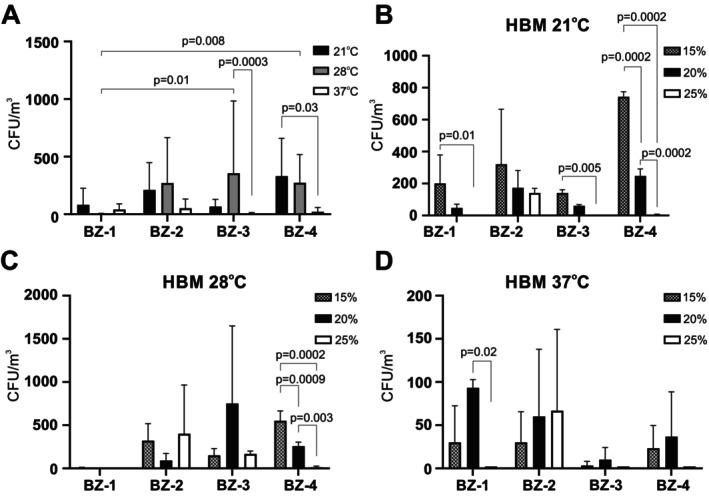
Presence of halophilic microorganisms in the air at the four sampling sites. Colonies of halophilic microorganisms collected from the air at the indicated sampling sites and grown on HBM agar containing 15%, 20% or 25% NaCl were counted after 3 months incubation at 21°C, 28°C or 37°C. The values shown in panel A represent the average CFU counts ± standard deviations grown at all salinities at 21°C (black bars), 28°C (grey bars) or 37°C (white bars), with three samples counted for each condition. Values in panels B‐D represent the average CFU counts ± standard deviations grown with 15% (dotted bars), 20% (black bars) or 25% NaCl (white bars) at 21°C (B), 28°C (C) or 37°C (D).

As expected, very few halophilic microorganisms were collected on HBM agar plates from BZ‐1, especially at 37°C and almost none at 28°C (Figure 5A). Most halophiles from BZ‐2, BZ‐3 and BZ‐4 were recovered on HBM agar plates incubated at 28°C, followed by 21°C and then 37°C (Figure 5A). When the numbers of CFU of the halophiles were calculated according to the salinity, most halophiles grew at 15% or 20% NaCl (Figure 5 B‐D), especially in BZ‐1, BZ‐3 and BZ‐4 at 21°C (Figure 5B). For BZ‐4, halophiles grew mostly on HBM agar with 15% or 20% NaCl, except for BZ‐2, where most grew at 25% (Figure 5D). While halophilic archaea were isolated from all four sites, 16S rRNA gene sequencing revealed only two different species (*Halalkalicoccus subterraneus* and *Halococcus hamelinensis* ) (Table 1).

**TABLE 1:** Halophilic microbial species isolated from the air of the Bochnia Salt Mine in the winter season.

Halophilic species	Accession number	% identity^a^	Domain	Media (% NaCl)^b^
*Halalcalicoccus subterraneus* GSM28^T^	MG097856	99.87	Archaea	20%
*Halococcus hamelinensis* 100A6^T^		99.76	Archaea	15%
*Lentibacillus persicus* AMB31^T^	FN376846	99.90	Bacteria	20%
*Piscibacillus halophilus* *HS224* ^*T*^	FM864227	100	Bacteria	20%
*Alkalibacillus halophilus* *YIM 012* ^*T*^	DQ359731	100	Bacteria	20%
*Virgibacillus kapii* *KN3‐8‐4* ^*T*^	LC041942	100	Bacteria	20%
*Salinicoccus halodurans* *CGMCC 1.6501* ^*T*^	DQ989633	98.67	Bacteria	20%
*Chromohalobacter canadensis* *ATCC 43984* ^*T*^	AJ295143	98.26	Bacteria	20%
*Lentibacillus amyloliquefaciens* *LAM0015* ^*T*^	CP013862	99.79	Bacteria	25%
*Alkalibacillus almallahensis* *S1LM8* ^*T*^	MF179558	100	Bacteria	20%

^a^ Percent of 16S rRNA gene identity with the closest species with validly published names using the online EzBioCloud database (Yoon et al. 2017).

^b^ Percent of salinity at which the strains were cultured on HBM agar.

### 3.4 Non‐Halophilic Microorganism Composition and Identification

**FIGURE 6:**
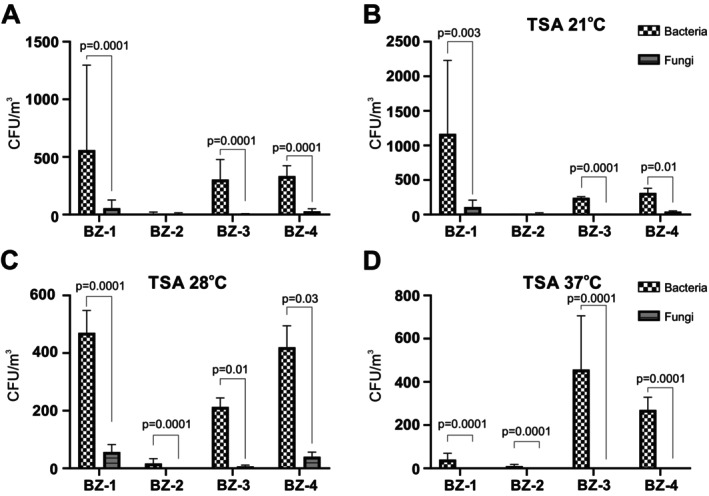
Presence of non‐halophilic fungi and bacteria in the winter air at the four sampling sites (BZ‐1 to BZ‐4). Colonies of non‐halophilic bacteria (dotted bars) and fungi (hatched bars) collected from the air at the four indicated sampling sites were counted after growth for 10 days on TSA at 21°C, 28°C or 37°C. The values shown represent the average CFU counts ± standard deviations at all temperatures (A), or at 21°C (B), 28°C (C) or 37°C (D), with three samples counted for each condition.

When the samples collected on TSA were examined for the presence of mould fungi and bacteria, the bacteria were found to dominate in BZ‐1, BZ‐3 and BZ‐4. There were only very few bacteria and fungi cultured from BZ‐2 (Figure 6A). When these samples were further subdivided according to their growth temperature, bacteria significantly dominated at 21°C in BZ‐1, BZ‐3 and BZ‐4 (Figure 6B) and at all four sites at 28°C (Figure 6C) and 37°C (Figure 6D). Eleven different bacterial species were identified using the VITEK system and characterised for the biochemical, metabolic and antibiotic resistance profile (Table S1). For seven isolates, the bacterial species could be unambiguously identified, while for four isolates, the bacteria could only be identified at the genus level (Table 2).

**TABLE 2:** Non‐halophilic bacterial species isolated from the air of the Bochnia Salt Mine.

Species	Characteristic
*Alicyclobacillus acidoterrestris*/*Alicyclobacillus acidocaldarius*	BCL
*Bacillus licheniformis*	BCL
*Bacillus subtilis*/*Bacillus amyloliquefaciens*/*Bacillus atrophaeus*	BCL
*Kocuria rhizophila*	GP
*Kocuria rosea*, *Dermacoccus nishinomiyaensis*/*Kytococcus sedentarius*	GP
*Kocuria varians*	GP
*Micrococcus luteus*	GP
*Micrococcus luteus*/*Micrococcus lylae*/*Granulicatella adiacens*	GP
*Sphingomonas paucimobilis*	GN
*Staphylococcus warneri*	GP
*Vagococcus fluvialis*	GP

Abbreviations: BCL, Spore Forming Bacilli; GN, Gram Negative; GP, Gram Positive.

### 3.5 Comparison of the Numbers of Halophilic and Non‐Halophilic Microorganisms Between Summer and Winter

**FIGURE 7:**
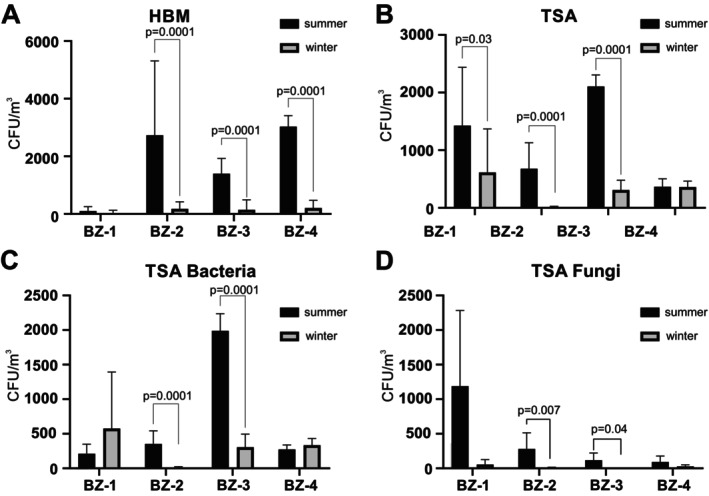
Seasonal variation in densities of halophilic microorganisms and non‐halophilic fungi and bacteria in the air at the four sampling sites (BZ‐1 to BZ‐4). The numbers of colonies of halophilic microorganisms (A), non‐halophilic microorganisms (B), bacteria (C) and fungi (D) in the air were compared between the summer season (black bars) and the winter season (grey bars). The values shown represent the average CFU counts ± standard deviations at all temperatures, with three samples counted for each condition.

Substantially more halophilic archaea were collected in the summer season compared to the winter season (Figure 7A). This was observed for all sampling sites, but in particular for BZ‐2 and BZ‐4. Non‐halophilic microorganisms were also generally more frequently isolated in the summer than in the winter except for BZ‐4 where no difference between the two seasons was found (Figure 7B). This difference between seasons was also reflected in the sites BZ‐2 and BZ‐3, where the number of bacteria significantly dominated in the summer season compared to the winter season (Figure 7C).

Finally, high numbers of fungi were isolated at the mine entrance in the summer and much less in the winter (Figure 7D). At the other sites, almost no fungi were collected in the winter, while significantly more were collected in the summer, albeit at lower numbers than at the mine entry.

## 4 Discussion

Our study confirms and extends earlier work that first detected viable halophilic archaea in summer air masses of the Bochnia Salt Mine (Puławska et al. 2025). We now show that winter air, being both cooler and drier, contains significantly fewer airborne halophiles, in parallel with decreases in aerosol Na^+^, Ca^2+^, Mg^2+^, Cl^−^ and SO_4_^2−^. Across all underground sites (excluding the entrance), winter halophile abundance was at least 13‐fold lower, and the richness of halophilic archaea species declined from seven to two taxa.

This seasonal pattern likely reflects the unique microclimatic dynamics of the historic Bochnia Salt Mine, which is relatively shallow (70–300 m) and no longer in industrial use. Contemporary salt mines are not only much deeper but also far more extensive, which favours environmental equilibration and leads to stable, dry, quasi‐isothermal conditions at depth and sufficient distance from ventilation shafts (Gębarowska et al. 2018; Khalid 2010; Naseem et al. 2020; Szlązak et al. 2000; Sambeek 2012; Puławska, Manecki, Flasza, Waluś, and Wojtowicz 2021). Although specific humidity data are lacking, the presumed dryness of active salt mines such as Winsford (UK) and the New Mexico mine (USA) may help explain why three studies, described by McGenity et al. (2000), failed to culture halophilic microorganisms despite extensive air sampling. Bochnia's limited size and depth make it more sensitive to surface‐driven fluctuations, providing a rare opportunity to study airborne halophiles under naturally variable salinity and humidity conditions.

Water availability, mediated directly through liquid water or indirectly via atmospheric humidity, is a fundamental ecological constraint for extremophilic microorganisms. Numerous studies in hyper‐arid regions, including the Atacama Desert, have shown that relative air humidity strongly correlates with microbial abundance and diversity in lithic habitats such as halite and gypcrete (Robinson et al. 2015; Azua‐Bustos et al. 2020; Meslier et al. 2018; Casero et al. 2020). In halite, high humidity promotes deliquescence, enabling the persistence of active microbial communities, whereas low humidity limits diversity and excludes more sensitive taxa (Robinson et al. 2015).

This ecological framework helps to explain our findings in the Bochnia Salt Mine, where summer air, characterised by higher humidity and greater salt aerosol content, supported significantly more abundant and diverse halophilic communities. In addition to a 13‐fold lower halophile density in the winter compared to the summer, only two archaeal species, *Hac. subterraneus* and *Hcc. hamelinensis*, were detected in the winter, compared to seven species identified in the summer (Puławska et al. 2025). Both *Hac. subterraneus* and *Hcc. hamelinensis* were also present in the summer. The reasons for their persistence in the winter are not entirely clear, but may reflect their resilience to limited moisture availability and other unfavourable conditions prevailing in the winter. *Hac. subterraneus* has indeed been shown to be relatively tolerant to osmotic stress and depends less on certain types of salts for growth, such as Mg ions (Chen et al. 2019), which were absent in the air samples during the winter season. The genome analysis of *Hcc. hamelinensis* also suggests pathways involved in osmoprotection for this halophile (Gudhka et al. 2015). In contrast to the archaea, the diversity of halophilic bacteria in the winter season was higher than that of halophilic archaea.

Regarding the non‐halophilic bacterial species, 11 species were identified in the winter air samples. This species diversity is consistent with previous observations that bacterial diversity remains relatively stable across seasons in Bochnia Salt Mine (Frączek et al. 2013). These results suggest that halophilic archaea are more sensitive to seasonal microclimatic fluctuations than non‐halophilic bacteria. Interactions between such bacteria and the halophilic archaea, especially *Hcc. hamelinensis*, may occur, as this organism is known to be associated with highly diverse microbial communities (Burns et al. 2004), and genome analyses of *Hcc. hamelinensis* revealed evidence of horizontal gene transfer from various bacterial lineages (Gudhka et al. 2015). During the air sampling we have not detected any viable pathogenic microbes. All identified non‐halophilic and halophilic microorganisms are known to be non‐pathogenic.

TSP measurements revealed clear seasonal and anthropogenic patterns. During the summer, when visitor frequency was highest (497 vs. 104 in the winter), both TSP levels and the abundance of non‐halophilic microorganisms increased, with a positive correlation between them (Puławska et al. 2025). The TSP fraction contained human‐derived components, such as rolled skin flakes and textile fibres (Puławska, Manecki, Flasza, and Styszko 2021), indicating that coarse, anthropogenic particles likely act as carriers for these taxa. In contrast, halophilic microorganisms showed no correlation with TSP in either season, supporting our earlier conclusion (Puławska et al. 2025) that halophilic taxa are primarily associated with the liquid (droplet) aerosol fraction formed under high relative humidity conditions.

Our microclimatic measurements indicated that the RH in the Bochnia Salt Mine is consistently higher in the summer (approximately 65%–77% RH) than in the winter (approximately 42%–63% RH). This seasonal range brackets the deliquescence threshold of halite (75% ± 2% RH at approximately 20°C), above which salt absorbs moisture and forms thin brine films (Tang and Munkelwitz 1993). In the summer, the influx of warm, humid surface air drives halite deliquescence, enhances salt dissolution and generates saline aerosols (Puławska, Manecki, Flasza, and Styszko 2021; Seinfeld and Pandis 2006), which most likely act as both transport vehicles and microhabitats for airborne halophiles. In the winter, reduced RH suppresses deliquescence and aerosol formation, curtailing halophile dispersal and exposing them to osmotic stress to which *Hac. subterraneus* and *Hcc. hamelinensis*, both detected in the winter, appear to be less sensitive than other halophilic archaea detected in the salt mine air only during the summer.

The depth transect illustrates this mechanism most clearly. At the furthest, undisturbed site BZ‐4 (2671 m from the entrance, 230 m deep), the summer RH reached 77%, continuous brine aerosols accumulated, and dense halophilic communities were recovered from the air (≥ 10^3^ CFU/m^3^); (Puławska et al. 2025). When RH fell to 63% in the winter, the combined Na^+^ + Cl^−^ aerosol mass decreased from 2665 μg/m^3^ to 1280 μg/m^3^ (~52% drop), and culturable halophiles plummeted from 7164 CFU/m^3^ in the summer to 528 CFU/m^3^ in the winter (~93% drop). A decrease was also observed for non‐halophilic microorganisms in sampling sites BZ‐1, BZ‐2 and BZ‐3, where the global number of CFU/m^3^ in the summer dropped from 4210 to 938 in the winter (~78% drop). No decrease in the numbers of non‐halophilic microorganisms was observed in BZ‐4, which indicates a much lower dependence on seasonal fluctuations for these microorganisms.

Seasonal contrasts were modulated by functional zoning. In the undisturbed mid‐transect tunnel BZ‐2, airborne halophiles persisted even in the winter, suggesting that capillary condensation or microscale brines can survive below the bulk deliquescence point. By contrast, the heavily trafficked Ważyn Chamber (BZ‐3) was dominated all year‐round by visitor‐borne, non‐halophilic bacteria, as was also observed in a previous study in the Bochnia Salt Mine (Frączek et al. 2013). Their abundance increased notably during the summer months, likely reflecting elevated visitor numbers (Frączek et al. 2013; Puławska, Manecki, Flasza, and Styszko 2021) and are therefore likely transient, as proposed by Górny et al. (2020), who noticed that after the therapeutic activities the bacterial densities decreased in all seasons. Anthropogenic inputs thus restructure non‐halophilic assemblages (Frączek et al. 2013; Gębarowska et al. 2018; Myszkowska et al. 2019), whereas halophilic archaea continue to track the physicochemical envelope set by salt hygroscopicity. Taken together, these observations suggest that seasonal differences in rock–aerosol interactions may play a key role in shaping halophile community dynamics in the air of the Bochnia Salt Mine. Although not directly investigated in this study, periodic deliquescence and efflorescence of halite crystals likely act as transient reservoirs that release halophilic microorganisms into the mine atmosphere. This process is being explored in our ongoing study of microbial communities on rock surfaces within the Bochnia Salt Mine.

Beyond the seasonal scope of the present study, long‐term climatic trends and engineering interventions such as modified ventilation or the introduction of air‐conditioning systems may strongly influence microbial balance within subterranean salt environments. Progressive surface warming and altered atmospheric humidity may affect mine ventilation dynamics, altering internal microclimates and water vapour exchange (Szlązak et al. 2000; Khalid 2010). Likewise, climate‐control systems implemented to maintain constant temperature and humidity for visitor comfort or heritage preservation may unintentionally shift physicochemical parameters that govern microbial survival. Sustained reductions in relative humidity could suppress halite deliquescence and limit halophilic diversity, whereas more humid conditions might promote the persistence and proliferation of halophilic microorganisms, potentially modifying community composition and competitive interactions with non‐halophilic and anthropogenic taxa. These scenarios underscore the importance of long‐term, multidisciplinary monitoring that couples climatic, physical and biological data to evaluate the resilience and ecological balance of subterranean microbiomes.

In conclusion, our findings thus indicate that airborne halophilic archaea respond sensitively to microclimatic water activity, suggesting that relative humidity is a key factor controlling their occurrence in subterranean salt environments. In contrast, the composition of airborne non‐halophilic bacterial communities is shaped primarily by human occupancy, especially in the summer. To confirm the indigenous origin of halophiles and to rule out the salt rock as a reservoir for non‐halophilic taxa, ongoing work includes cultivation surveys of the lithic matrix itself.

The findings of this study expand our understanding of microbial ecology in subterranean salt environments and underline the sensitivity of these systems to both climatic and anthropogenic pressures. Beyond their scientific relevance, the results provide a framework for integrating microbial observations into microclimate management strategies aimed at maintaining environmental stability and preserving the unique biosphere of heritage salt mines. Similar concerns have been raised for other salt‐heritage sites, such as the Hallstatt mine in Austria, where halophilic microorganisms were identified as contributors to biodeterioration of a 3100‐year‐old wooden staircase (Piñar et al. 2016). These parallels highlight the importance of incorporating microbial monitoring into long‐term mine management and conservation strategies, ensuring that ventilation and climate‐control practices maintain both structural integrity and ecological stability in heritage salt environments to prevent microbially driven degradation in salt environments. Future studies should further explore the connections between airborne and rock‐surface communities and assess how long‐term climatic or engineering changes may affect microbial resilience in these confined ecosystems.

Limitations of the study include the fact that we only collected microorganisms culturable either on HBM or TSA, and viable non‐culturable organisms or microorganisms culturable in other conditions, such as actinomycetes and viruses, were not included in this study. This study does therefore not fully describe the microbial diversity in the Bochnia Salt Mine air. However, we deliberately limited the study to culturable microorganisms in order to compare the winter with the summer seasons, based on our previous study which also only included culturable microorganisms (Puławska et al. 2025). Furthermore, this allows us to study the biology and potential biotechnological applications of these microorganisms in future work. An additional limitation of this study is that it only describes culturable microorganisms between seasons within the same cycle and in the same salt mine. Moreover, our sampling design only contrasted two seasonal extremes (summer and winter) within a single annual cycle in one salt mine; transitional seasons (spring and autumn) and interannual variability were not captured and should be addressed by future multi‐season and multi‐year monitoring campaigns. Future work should also test whether the observed microbial shifts are consistent across different salt mines used for recreational and halotherapeutic purposes and combine cultivation‐based surveys with metagenomic approaches to better assess biodiversity and seasonal shifts. It is at present not known whether the profound differences in haloarchaeal densities and species richness in the air between summer and winter may have an effect on the therapeutic efficacy of halotherapy. We are currently comparatively analyzing the effect of the various isolated strains on the responses of human innate immune cells, known to be central players in immune modulation. It would also be interesting to design randomized controlled clinical trials comparing the halotherapeutic efficacy between winter and summer.

## Author Contributions

Conceptualization: A.P., M.K.‐K., M.M. Funding acquisition: A.P., M.M., L.A., C.L., M.K.‐K. Sampling A.P., M.R., J.K., M.K.‐K. Performed the experiments: A.P., M.K.‐K., M.R., D.D. Analysed the data: A.P., M.K.‐K., L.A., C.E., M.M., C.L. Project administration: A.P. Visualisation: A.P., M.R., K.K. Contributed reagents/materials: A.P., M.K.‐K. Writing – original draft preparation: M.K.‐K., A.P., C.L. Writing – review and editing: A.P., M.K.‐K., M.M., C.L. All authors read and approved the manuscript.

## Funding

This work was supported by Narodowe Centrum Nauki (2021/41/N/ST10/02751); Narodowa Agencja Wymiany Akademickiej (BPN/ULM/2023/1/00090/U/00001); ERDF – European Regional Development Fund (COMPETE 2020); FCT ‐ Foundation for Science and Technology: UIDB/04539/2020, UIDP/04539/2020, LA/P/0058/2020.

## Conflicts of Interest

The authors declare no conflicts of interest.

## Supporting information


**Table S1:** Biochemical characteristics of Gram‐positive (GP), Gram‐negative (GN) and Gram‐positive spore‐forming bacilli microorganisms (BCL), isolated from the air of Bochnia Salt Mine, identified by VITEK‐2 system.

## Data Availability

The data that support the findings of this study are available on request from the corresponding author. The data are not publicly available due to privacy or ethical restrictions.
