# Supplementary material for: Airborne Halophilic and Non‐Halophilic Microbial Communities in an Underground Salt Mine Affected by Seasonal Environmental Fluctuations
Source: Environ Microbiol Rep. 2026 Feb 18;18(1):e70293. doi: 10.1111/1758-2229.70293 (PMC12914699; doi:10.1111/1758-2229.70293)
Supplement: Supplementary file 1 — Table S1: Biochemical characteristics of Gram‐positive (GP), Gram‐negative (GN) and Gram‐positive spore‐forming bacilli microorganisms (BCL), isolated from the air of Bochnia Salt Mine, identified by VITEK‐2 system. [file EMI4-18-e70293-s001.docx]

***Table S1: Biochemical characteristics of Gram-positive (GP), Gram-positive spore-forming bacilli* (BCL)*, and Gram-negative* (GN) *microorganisms isolated from the air of Bochnia Salt Mine, identified by VITEK-2 system***

|  | | **GP** | | | | | | |
| --- | --- | --- | --- | --- | --- | --- | --- | --- |
|  |  | **Genus** | *Kocuria* | | | *Micrococcus* | *Vagococcus* | *Staphylococcus* |
|  |  | **Species** | *varians* | *rhizophila* | *rosea* | *luteus* | *fluvalis* | *warneri* |
|  |  | **Sample** | 1D | 9D | 13D | 11D | 18D | 20D |
| **SUGAR BREAKDOWN** | D-AMYGDALIN | *AMY* | - | - | - | - | **+** | - |
|  | D-XYLOSE | *dXYL* | - | - | - | - | - | - |
|  | CYCLODEXTRIN | *CDEX* | - | - | - | - | **+** | - |
|  | D-SORBITOL | *dSOR* | - | - | - | - | **+** | - |
|  | D-GALACTOSE | *dGAL* | - | - | - | - | - | - |
|  | D-RIBOSE | *dRIB* | - | - | - | - | **+** | - |
|  | LACTOSE | *LAC* | - | - | - | - | - | - |
|  | N-ACETYL-D-GLUCOSAMINE | *NAG* | - | - | - | - | **+** | - |
|  | D-MALTOSE | *dMAL* | - | - | - | - | **+** | - |
|  | D-MANNITOL | *dMAN* | - | - | - | - | **+** | - |
|  | D-MANNOSE | *dMNE* | - | - | - | - | **+** | - |
|  | METHYL-B-D-GLUCOPYRANOSIDE | *MBdG* | - | - | - | - | - | - |
|  | PULLULAN | *PUL* | - | - | - | - | - | - |
|  | D-RAFFINOSE | *dRAF* | - | - | - | - | - | - |
|  | SACCHAROSE/SUCROSE | *SAC* | - | **+** | - | - | **+** | **+** |
|  | D-TREHALOSE | *dTRE* | - | - | - | - | **+** | **+** |
| **ENZYMATIC ACTIVITY** | PHOSPHATIDYLINOSITOL PHOSPHOLIPASE | *CPIPLC* | - | - | - | - | - | - |
|  | ARGININE DIHYDROLASE 1 | *ADH1* | **+** | **+** | - | - | - | **+** |
|  | BETA-GALACTOSIDASE | *BGAL* | - | - | - | - | - | - |
|  | ALPHA-GLUCOSIDASE | *AGLU* | - | **+** | - | **+** | **+** | - |
|  | ALA-PHE-PRO ARYLAMIDASE | *APPA* | - | - | - | **+** | - | - |
|  | L-ASPA21ATE ARYLAMIDASE | *AspA* | - | - | - | - | **+** | - |
|  | BETA GALACTOPYRANOSIDASE | *BGAR* | - | - | - | - | - | - |
|  | ALPHA-MANNOSIDASE | *AMAN* | - | - | - | - | - | - |
|  | PHOSPHATASE | *PHOS* | - | - | - | - | - | - |
|  | LEUCINE ARYLAMIDASE | *LeuA* | - | **+** | - | **+** | - | - |
|  | L-PROLINE ARYLAMIDASE | *ProA* | - | - | **+** | **+** | **+** | - |
|  | BETA GLUCURONIDASE | *BGURr* | - | - | - | - | - | **+** |
|  | ALPHA-GALACTOSIDASE | *AGAL* | - | - | - | - | - | - |
|  | L-PYRROLYDONYL-ARYLAMIDASE | *PyrA* | - | **+** | - | **+** | **+** | - |
|  | BETA-GLUCURONIDASE | *BGUR* | - | - | - | - | - | - |
|  | ALANINE ARYLAMIDASE | *AlaA* | **+** | **+** | **+** | **+** | - | - |
|  | TYROSINE ARYLAMIDASE | *TyrA* | - | - | - | **+** | **+** | - |
|  | UREASE | *URE* | **+** | - | - | - | - | - |
|  | SALICIN | *SAL* | - | - | - | - | **+** | - |
|  | ARGININE DIHYDROLASE 2 | *ADH2s* | - | - | - | - | - | - |
| **ANTIBIOTIC RESISTANCE** | POLYMIXIN B RESISTANCE | POLYB | - | - | - | - | **+** | - |
|  | BACITRACIN RESISTANCE | BACI | - | - | - | - | **+** | - |
|  | NOVOBIOCIN RESISTANCE | *NOVO* | - | - | - | - | **+** | - |
|  | O/129 RESISTANCE (COMP.VIBRIO.) | *O129R* | - | - | - | - | - | **+** |
|  | OPTOCHIN RESISTANCE | *OPTO* | - | **+** | - | - | **+** | **+** |
| **OTHER** | L-LACTATE ALKALINIZATION | *ILATk* | - | **+** | - | **+** | - | **+** |
|  | GROWTH IN 6.5% NACL | *NC6.5* | - | - | - | - | - | **+** |

|  | | **BCL** | | | |
| --- | --- | --- | --- | --- | --- |
|  |  | **Genus** | *Bacillus* | | *Alicylobacillus* |
|  |  | **Species** | *lichenformis* | *subtilis* | *acidoterrestris* |
|  |  | **Sample** | 2D | 17D | 3D |
| **SUGAR BREAKDOWN** | D-GALACTOSE | *DGAL* | - | - | - |
|  | GLYCOGEN | *GLYG* | **+** | - | - |
|  | MYO-INOSITOL | *INO* | **+** | - | - |
|  | METHYL-D-XYLOSIDE | *MDX* | - | - | - |
|  | MALTOTRIOSE | *MTE* | **+** | - | - |
|  | D-MANNITOL | *DMAN* | **+** | **+** | - |
|  | D-MANNOSE | *DMNE* | - | **+** | - |
|  | D-MELEZITOSE | *DMLZ* | - | - | - |
|  | PALATINOSE | *PLE* | - | - | - |
|  | L-RHAMNOSE | *IRHA* | - | - | - |
|  | PYRUVATE | *PVATE* | **+** | **+** | - |
|  | D-TAGATOSE | *DTAG* | **+** | - | - |
|  | D-TREHALOSE | *DTRE* | **+** | **+** | - |
|  | INULIN | *INU* | - | **+** | - |
|  | D-GLUCOSE | *DGLU* | **+** | **+** | - |
|  | D-RIBOSE | *DRIB* | **+** | **+** | - |
| **ENZYMATIC ACTIVITY** | BETA-XYLOSIDASE | *BXYL* | - | **+** | - |
|  | L-LYSINE-ARYLAMIDASE | *LYSA* | - | - | **+** |
|  | L-ASPA21ATE ARYLAMIDASE | *ASPA* | - | - | - |
|  | EUCINE-ARYLAMIDASE | *LEUA* | **+** | **+** | **+** |
|  | PHENYLALANINE ARYLAMIDASE | *PHEA* | **+** | **+** | **+** |
|  | L-PROLINE ARYLAMIDASE | *PROA* | - | - | - |
|  | BETA-GALACTOSIDASE | *BGAL* | - | **+** | - |
|  | L-PYRROLYDONYL-ARYLAMIDASE | *PYRA* | **+** | **+** | - |
|  | ALPHA-GALACTOSIDASE | *AGAL* | - | **+** | - |
|  | ALANINE ARYLAMIDASE | *ALAA* | - | - | **+** |
|  | TYROSINE ARYLAMIDASE | *TYRA* | **+** | **+** | **+** |
|  | ETA-N-ACETYL-GLUCOSAMINIDASE | *BNAG* | - | - | - |
|  | ALA-PHE-PRO ARYLAMIDASE | *APPA* | **+** | - | **+** |
|  | CYCLODEXTRIN | *CDEX* | **+** | - | - |
|  | ALPHA-MANNOSIDASE | *AMAN* | - | - | - |
|  | GLYCINE ARYLAMIDASE | *GLYA* | - | **+** | - |
|  | N-ACETYL-D-GLUCOSAMINE | *NAG* | - | - | - |
|  | BETA-GLUCOSIDASE | *BGLU* | - | **+** | - |
|  | BETA-MANNOSIDASE | *BMAN* | - | **+** | - |
|  | ALPHA-GLUCOSIDASE | *AGLU* | - | **+** | - |
| **ANTIBIOTIC RESISTANCE** | KANAMYCIN RESISTANCE | *KAN* | - | - | - |
|  | OLEANDOMYCIN RESISTANCE | *OLD* | - | - | - |
|  | POLYMIXIN_E RESISTANCE | *POLYB_R* | **+** | - | - |
| **OTHER** | METHYL-A-D-GLUCOPYRANOSIDE ACIDIFICATION | *PHC* | - | - | - |
|  | ELLMAN | *MDG* | **+** | - | - |
|  | PHOSPHORYL CHOLINE | *ELLM* | - | - | - |
|  | PUTRESCINE ASSIMILATION | *PSCNA* | **+** |  | - |
|  | GROWTH IN 6.5% NAC | *NAC 6.5%* | **+** | **+** | - |
|  | ESCULIN HYDROLYSIS | *ESC* | **+** | **+** | - |
|  | TETRAZOLIUM RED | *TTZ* | **+** | - | - |

|  | | **GN** | | |
| --- | --- | --- | --- | --- |
|  |  | **Genus** | *Sphingomonas* | |
|  |  | **Species** | *paucimobilis* | *paucimobilis* |
|  |  | **Sample** | 5D | 15D |
| **SUGAR BREAKDOWN** | ADONITOL | *ADO* | - | - |
|  | L-ARABITOL | *IARL* | - | - |
|  | D-CELLOBIOSE | *dCEL* | **+** | - |
|  | D-GLUCOSE | *dGLU* | **+** | - |
|  | FERMENTATION/ GLUCOSE | *OFF* | - | - |
|  | D-MALTOSE | *dMAL* | **+** | - |
|  | D-MANNITOL | *dMAN* | - | - |
|  | D-MANNOSE | *dMNE* | **+** | - |
|  | D-SORBITOL | *dSOR* | - | - |
|  | SACCHAROSE/SUCROSE | *SAC* | **+** | - |
|  | D-TAGATOSE | *dTAG* | - | - |
|  | D-TREHALOSE | *dTRE* | **+** | - |
|  | MALONATE | *MNT* | - | - |
|  | 5-KETO-D-GLUCONATE | *5KG* | - | - |
| **ENZYMATIC ACTIVITY** | ALA-PHE-PRO-ARYLAMIDASE | *APPA* | **+** | **+** |
|  | L-PYRROLYDONYL-ARYLAMIDASE | *PyrA* | **+** | **+** |
|  | BETA-GALACTOSIDASE | *BGAL* | **+** | - |
|  | H2S PRODUCTION | *H2S* | - | - |
|  | BETA-N-ACETYL-GLUCOSAMINIDASE | *BNAG* | **+** | - |
|  | GLUTAMYL ARYLAMIDASE PNA | *AGLTp* | - | - |
|  | GAMMA-GLUTAMYL-TRANSFERASE | *GGT* | - | - |
|  | BETA-GLUCOSIDASE | *BGLU* | **+** | - |
|  | BETA-XYLOSIDASE | *BXYL* | - | - |
|  | BETA-ALANINE ARYLAMIDASE PNA | *BAIap* | **+** | - |
|  | L-PROLINE ARYLAMIDASE | *ProA* | **+** | - |
|  | LIPASE | *LIP* | - | - |
|  | PALATINOSE | *PLE* | **+** | - |
|  | TYROSINE ARYLAMIDASE | *TyrA* | **+** | **+** |
|  | UREASE | *URE* | - | - |
|  | ALPHA-GLUCOSIDASE | *AGLU* | **+** | - |
|  | BETA-N-ACETYL-GALACTOSAMINIDASE | *NAGA* | - | - |
|  | ALPHA-GALACTOSIDASE | *AGAL* | - | - |
|  | PHOSPHATASE | *PHOS* | - | - |
|  | GLYCINE ARYLAMIDASE | *GlyA* | **+** | - |
|  | ORNITHINE DECARBOXYLASE | *ODC* | - | - |
|  | LYSINE DECARBOXYLASE | *LDC* | - | - |
|  | DECARBOXYLASE BASE | *0DEC* | - | - |
|  | BETA-GLUCURONIDASE | *BGUR* | - | - |
|  | GLU-GLY-ARG-ARYLAMIDASE | *GGAA* | - | **+** |
| **ANTIBIOTIC RESISTANCE** | O/129 RESISTANCE (COMP.VIBRIO.) | *O129R* | - | - |
| **OTHER** | CITRATE (SODIUM) | *CIT* | - | - |
|  | L-LACTATE ALKALINIZATION | *ILATk* | **+** | - |
|  | SUCCINATE ALKALINIZATION | *SUCT* | - | - |
|  | L-HISTIDINE ASSIMILATION | *IHISa* | - | - |
|  | COUMARATE | *CMT* | - | - |
|  | L-MALATE ASSIMILATION | *IMLTa* | - | - |
